# Bergmann’s rule is a “just-so” story of human body size

**DOI:** 10.1186/s40101-022-00287-z

**Published:** 2022-04-12

**Authors:** Barry Bogin, Michael Hermanussen, Christiane Scheffler

**Affiliations:** 1 UCSD/Salk Center for Academic Research and Training in Anthropogeny (CARTA), San Diego, USA; 2 School of Sport, Exercise & Health Sciences, Loughborough University, Loughborough, UK; 3 Aschauhof 3, 24340 Altenhof, Germany; 4 Institute of Biochemistry and Biology, Human Biology, University of Potsdam, Potsdam, Germany

**Keywords:** Developmental plasticity, SEPE, Body size, Body shape

## Abstract

Carl Bergmann was an astute naturalist and physiologist. His ideas about animal size and shape were important advances in the pre-Darwinian nineteenth century. Bergmann’s rule claims that that in cold climates, large body mass increases the ratio of volume-to-surface area and provides for maximum metabolic heat retention in mammals and birds. Conversely, in warmer temperatures, smaller body mass increases surface area relative to volume and allows for greater heat loss. For humans, we now know that body size and shape are regulated more by social-economic-political-emotional (SEPE) factors as well as nutrition-infection interactions. Temperature has virtually no effect. Bergmann’s rule is a “just-so” story and should be relegated to teaching and scholarship about the history of science. That “rule” is no longer acceptable science and has nothing to tell us about physiological anthropology.

## Introduction

The *Just So Stories* by Rudyard Kipling [1] are a type of origin myth, that is, they are “…fantastic accounts of how various features of animals came to be” [2]. With titles such as “How the camel got his hump” and “How the rhinoceros got his skin,” one interpretation of the phrase “just so” is that the stories lay out superficial and Lamarckian explanations for animal phenotypes [3] that delight children. Kipling defined the meaning of “just so” another way by explaining that they were bedtime tales told to his daughter; “...in the evening there were stories meant to put Effie to sleep, and you were not allowed to alter those by one single little word. They had to be told just so; or Effie would wake up and put back the missing sentence. So at last they came to be like charms, all three of them,—the whale tale, the camel tale, and the rhinoceros tale” (Kipling quoted by Karlin, 2015 [4]).

Educational researchers have long known that “Repetition aids learning complex information by increasing opportunities for the information to be encoded…” [5]. Anthropologists and religious leaders know that origin myths are repeated for a similar reason. These myths are culturally universal and have powerful influences on thinking and behavior for people of all ages [6, 7].

The purpose of this essay is to show that Bergmann’s rule for body size variation is an origin myth. It is a “just-so” story because it is a Lamarckian explanation for human phenotypes and because the Bergmann’s rule story has been repeated in virtually all biological anthropology and human physiology textbooks and has been told and retold in countless lectures. Combining its origin myth nature with its repetitive teaching has enshrined Bergmann’s rule in human biology scholarship. Here, it is shown that Bergmann’s rule was derived from pre-Darwinian biological philosophy, which was based on the biblical creation story of the Old Testament. When evaluated from the perspective of post-Darwinian science, there is no evidence that Bergmann’s rule applies to present-day humans and likely did not apply to any members of the genus *Homo*.

## Bergmann’s rule

Bergmann’s rule, and its corollary Allen’s rule, are usually considered to be examples of ecogeographic thermoregulation in relation to body shape. The physiological principle is that in cold climates, large body mass (Bergmann) and relatively short extremities (Allen) increase the ratio of volume-to-surface area and provide for a body shape that maximizes metabolic heat retention in a mammal. Conversely, in warmer climates, small body size with relative long extremities increases surface area relative to volume and allows for greater heat loss. Most of the assessments of these ecogeographic “rules” have been based on descriptive and correlational analyses of databases with information on body mass and extremity lengths (wings, limbs, tails) of homeothermic species of birds and mammals native to different latitudes [8, 9]. There is debate as to the following: (1) do the “rules” apply only between species, (2) only within species or within closely related species, or (3) if the rules apply at all. There are very few well-controlled experimental studies. One experimental study found that laboratory-bred mice raised in warmer temperature experienced greater growth of bone tissue chondrocytes [10]. The usual explanation for this is greater vascularization of the skeletal tissue, allowing for greater oxygen, nutrient perfusion, and cellular growth. Serrat and colleagues’ experimental research showed, however, that even in the absence of vasculature, in vitro culture of chondrocytes from mouse metatarsal bone had a positive correlation between environmental temperature with “…greater proliferation and extracellular matrix volume…” (2008, p. 19348). The authors’ interpretation is that rather than oxygen or nutrient delivery via the vascular, higher temperature, itself, is the stimulus for greater skeletal growth. These experimental findings, as well as statistical studies of human global databases and quasi-experiments with humans [11, 12], provide some support for Allen’s rule, but no support for Bergmann’s rule.

## Carl Bergmann: a pre-Darwinian biologist between creation and evolution

Carl Georg Lucas Christian Bergmann was a German anatomist and physiologist born on May 18, 1814, in Göttingen. He died on April 30, 1865, in Geneva. He was the son of the lawyer and professor Friedrich Christian Bergmann (1785–1845). After graduation in 1832 in Holzminden, he studied medicine and natural sciences at the universities of Göttingen and Würzburg. He worked as Privatdozent of medicine. In 1843, he was appointed associate professor in Göttingen. In 1846, he accompanied Sartorius von Waltershausen and Robert Bunsen on a research trip to Iceland. His most famous work is titled *On the Proportions of Heat Economy of Animals to their Size*^1^ where he discussed the relationship between heat balance and body size that has later been named Bergmann’s rule [13]. Bergmann’s book appeared 12 years before Darwin’s *On the Origin of Species* went on sale in November 1859. Bergmann’s considerations include creationistic ideas. Bergmann assessed God’s creation in a time before Darwinian evolutionary biology.

Bergmann knew about the work of Justus von Liebig, who systematically investigated the chemical foundation of the life processes, classified the constituents of foodstuffs, and tried to explain nutrition, metabolism, heat generation, and respiratory gas exchanges in chemical terms. Bergmann was aware that the heat production of warm-blooded animals is limited by their volume, and that all losses of heat depend on characteristics of the animal’s surfaces, temperature difference between the skin and air, and heat conduction. He concluded that the texture of the surface and the proportion of surface and volume of an animal are subject to the laws of physics. He ranked animal surfaces according to heat conductivity, with skin to water being most thermoconductive to skin to air and fur/plumage to air as least thermoconductive. He then ranked the animals known to him, from marine mammals and water-loving pachyderms, other terrestrial animals, and finally birds to determine the largest and the smallest possible sizes for species within God’s creation. Based on his considerations, Bergmann concluded that “…there must be a not crossable limit of smallness for endotherm (warm-blooded) animals caused by the proportion that the (heat producing) volume when decreasing in size, will decrease to a greater extent than its (heat loosing) surface.”

Bergmann particularly considered the extremes as he believed they represented the upper and lower possible limits for size in endotherms. Bergmann believed that hummingbirds are close to this lowest possible limit for size in endotherms. He then asked, “…whether in all places extremes in size have been reached or not…” and looked for the “largest and smallest homeotherm creatures.” He realized, “…that in the temperate and cold regions, extreme sizes even though possible according to this law, have not been reached…,” and he concluded that “…nature did not fully complete its limits which would have been offered according to this law…The largest animals of the cooler zones have for whatever reasons not been created.” Almost modern in his thinking, he further stated that it “…is not understandable, and rather looks like random particularly when remembering the large distribution of elephant-like and other very large animals *of earlier creations*” [emphasis added to note the pre-evolutionary thinking]. He formulated his hypothesis that “…if we could find two animal species which only differed in size…the geographic distribution of these two species should be relatively determined by their size; which absolutely taken would be their homeland, the smaller one should ask for a warmer, the larger one for a colder climate.” Bergmann found support for his hypothesis by comparing body size and wingspan of various birds known to him, of different species but the same family, and showed that in many species, the volume-to-surface ratio is associated with their geographic distribution. Using an approach that may be considered statistical, he found that a majority of species are subject to what is known today as Bergmann’s rule.

Bergmann’s rule is popular since more than 170 years and belongs to the list of what German-speaking people call “Lieschen Müller^2^ knowledge” — a type of “just-so” story. This type of knowledge refers to common concepts of understanding the world, which are transmitted early in life, often already at or even before school age. These concepts are beyond doubt and largely independent of later academic learning, scientific research, or personal experience. They are based on prevalent cultural perception. Bergmann’s rule is in this category of knowledge because it is included in almost every introductory textbook and university course related to animal physiology, ecology, and human biology. The same applies to its corollary, Allen’s rule, relating to limb proportions. Both “rules” are fundamental concepts, not only included in textbooks and taught in undergraduate and postgraduate anthropology and human biology courses but also assumed to be scientific truth by researchers conducting human ecogeographic research.

Bergmann’s 1848 book was published in an old-style German orthography that makes reading his book difficult, even for contemporary native speakers of German. There does not appear to be an English-language translation of the entire book. Consequently, few people have read the original, even those who cite the book in their own research and teaching. This has led to considerable misunderstanding about what Bergmann wrote in his book. As noted by Salewski and Watt [9], “…Bergmann himself never formulated an explicit rule…[and that researchers]…should either go back to the original publication (Bergmann 1847) when referring to it or simply not cite it at all.”

Similar admonitions apply to Allen’s rule. Joel Asaph Allen (1838–1921) published his most famous work in an English-language article titled, “The influence of physical conditions in the genesis of species” [14]. As noted by C. H. Smith, Allen’s article has been more cited than read, largely because it was published in an obscure journal that ceased operation after one year [15]. Allen was an American zoologist, the first president of the American Ornithologists’ Union, the first curator of birds and mammals at the American Museum of Natural History, and the first head of that museum’s Department of Ornithology. In his 1877 article, he proposed that in warm-blooded animals (endotherms), the ratio of limb length to total body size varies with climate. The limbs and tails of such species tend to be shorter in cold climates and longer in warmer environments [14]. The purpose of this variation is to increase surface area in hot climates to lose heat and minimize surface area in cold climates to conserve heat. Allen formulated this hypothesis 18 years after Darwin’s *Origin of Species* was published. Even so, Allen continued with Bergmann’s pre-evolutionary thinking. Allen cited Darwin’s *Origin of Species* and discussed natural selection but rejected it. Allen wrote, “…that other influences than natural selection operate powerfully in the differentiation of specific forms, and that geographical causes share more largely in the work than naturalists have heretofore been prepared to admit” [14]. Smith explained that Allen “…was a strong supporter of the late 19^th^ century American view that much evolutionary change came about through the direct action of the environment, and not natural selection” [15].

## Size and shape in living humans

One of the most common misunderstandings about Bergmann’s research is that he compared what he called “closely related species” living at different latitudes. Biological “species” was a poorly understood concept in 1848, but even so, Bergmann’s comparisons were *interspecific*. The application of Bergmann’s rule to human beings is *intraspecific*. In addition, while Bergmann analyzed more than 300 species of birds, belonging to 86 genera, he did not discuss human size. This was done some 100 years later by Derek Roberts (1925–2016) who was interested in the heat production of people according to the climate of their habitats [16]. The research of Roberts and other human biologists who followed is discussed below. Prior to that discussion, some background on human body size and shape is needed.

**Fig. 1:**
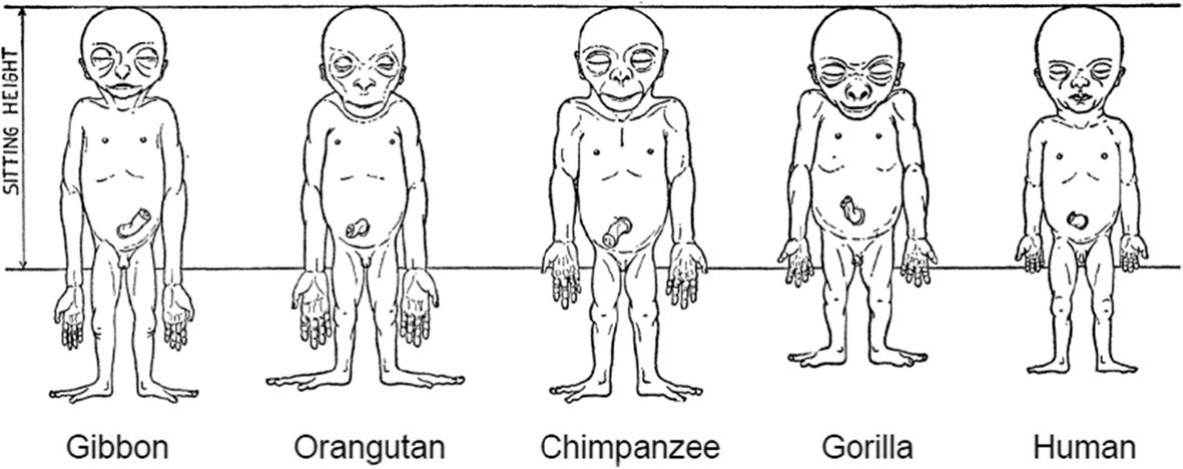
Schultz’s sketches of the body proportions of hominoid fetuses. The original legend for this figure states, “All the figures have the same sitting height. The human fetus is in the 4th month, the gorilla and the gibbon fetus correspond in development to the human fetus, but the chimpanzee and the orang fetus are slightly more advanced in their growth” [17], p. 465-466

The human adult phenotype is distinguished from that of the nonhuman primates by proportions of the arms and legs and by the size and shape of the cranium relative to the face and to total body length. These species-specific adult differences develop during the ontogeny of the individual. One classic example was provided by Adolf Schultz [17], who sketched the prenatal changes in body proportions of ape and human fetuses, reproduced here as Fig. 1. The human fetus “of the 4th month” has relatively shorter legs than the chimpanzee, orangutan, or gibbon. The accuracy of this difference assumes that Schultz estimated fetal development correctly for the nonhuman apes (see Fig. 1 legend). Another difference in proportion that may be noted in Fig. 1, but not mentioned by Schultz, is the size of the cranium relative to the face, which is larger in the human fetus than in the gorilla, chimpanzee, orangutan, or gibbon.

**Fig. 2:**
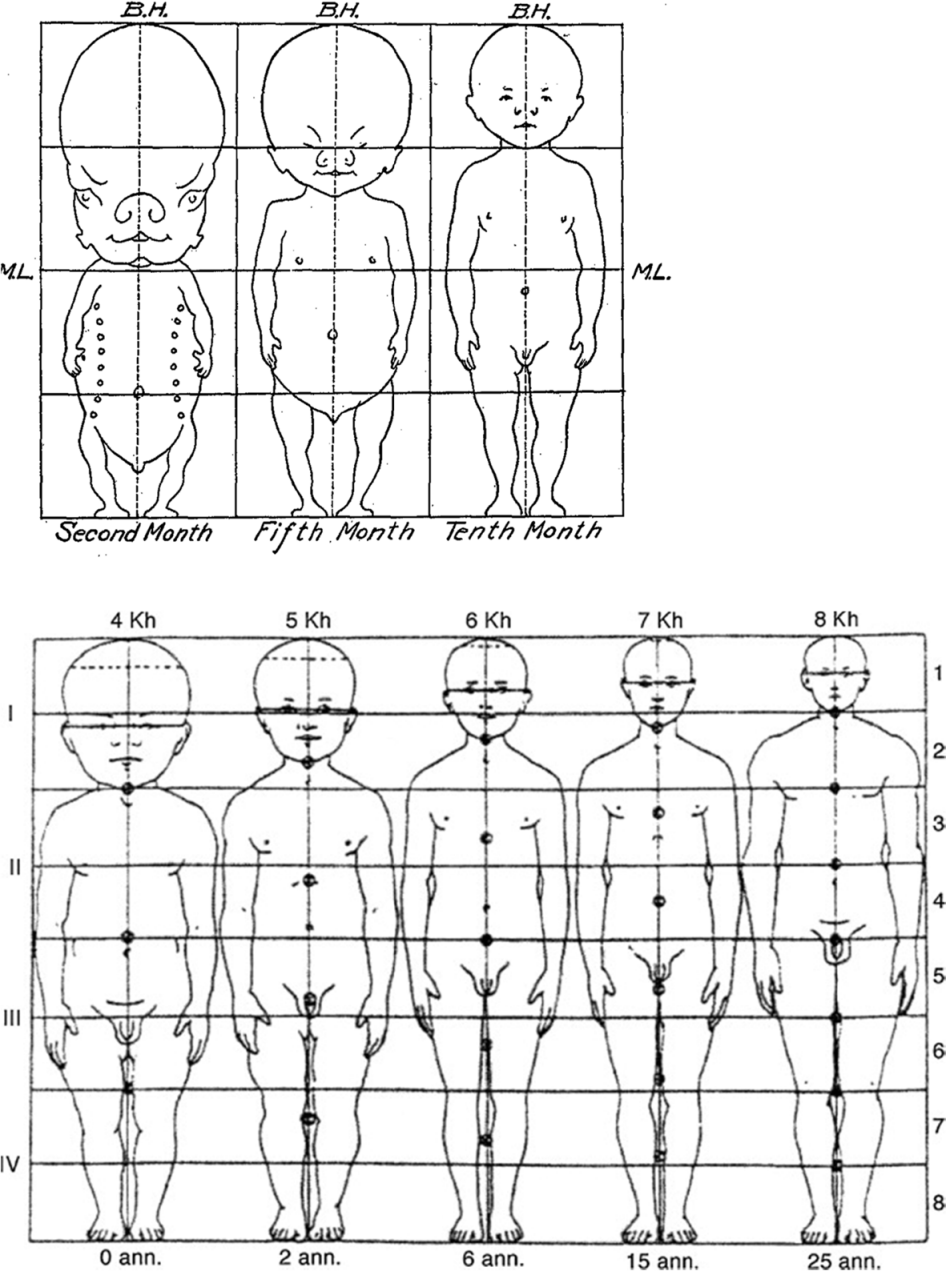
Changes in body proportions in fetal life (top illustration). *B.H.*, body height/length; *M.L.*, midline. Changes in body proportion during postnatal life (lower illustration). *kh*, head length as a percentage of total body length, 4 kh = 25%, 5 kh = 20%, etc.; *ann*., age in years; 0 *ann*., birth, etc. From Stratz [18], top illustration modified at http://www.neonatology.org/classics/hess1922/hess.3.html

The human fetus and neonate have a leg length that never exceeds ~33% of total body length, but legs grow relatively quickly after birth to become, on average, 48% of body length by adulthood. This remarkable change in body proportions was illustrated by Stratz [18], whose original illustration is reproduced here as Fig. 2. Leg length grows to 40% of total body length at age 2.5 years of age, 44% at age 5.5 years, and 46% by 8.5 years and achieves the adult value of 48% by age 11.5 years. These mean values are based on the worldwide growth compendia of Eveleth and Tanner [19, 20], combining data for males and females, multiple geographic regions, and all ethnicities. There are sex and geographic variations in body proportions, which are discussed below. The point to emphasize here is that the general pattern of human body shape development is a species-specific characteristic. Historical artwork, sculpture, and anatomical drawings from Renaissance Europe [21, 22] and pre-Columbian Mexico [23] show fundamental commonalities in the depiction of body shape of late-term fetuses, newborns and infants, children, adolescents, and adults.

**Fig. 3:**
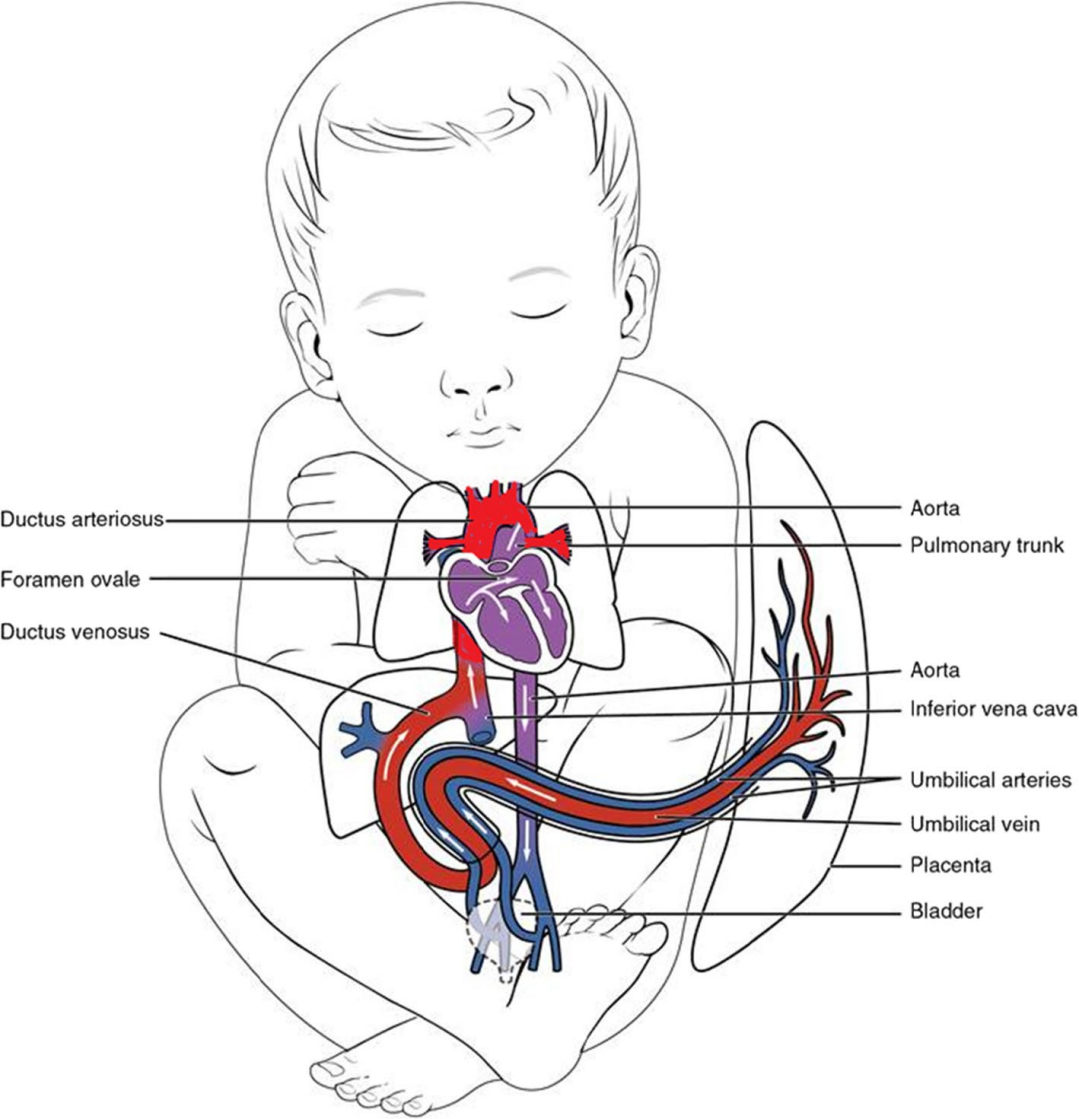
Human fetal circulation. The relative amount of oxygen in the fetal blood is greatest in the upper thorax, neck, and head, indicated by the red color of the vessels ascending from the heart. Blood flowing to the abdomen and legs is less well oxygenated, indicated by the violet color of the vessels descending from the heart. Vessels colored in blue indicate deoxygenated blood returning to the heart via the umbilical arteries (adapted from https://www.anatomynote.com/human-anatomy/infant-child-anatomy/fetal-newborn-baby-blood-vessels-circulation-diagram/)

The ape-human differences in the pattern of body proportion development from fetus to adult may be explained in large part by the evolution of human bipedalism and by the large and complex human brain. Bipedal locomotion places a biomechanical premium on having legs longer than arms. In contrast, the nonhuman apes practice brachiation, which places that premium on having arms longer than legs. The role of the brain in human biological and behavioral evolution is amply discussed elsewhere [24]. Human newborns are bigger brained than any of the apes (Table 1). Growing the human brain requires major inputs of energy, other nutrients, and oxygen — all supplied via the placenta and the fetal circulatory system. That system is designed to shunt more blood to the trunk and the head and less to the legs. In addition, blood in the fetal ascending aorta (toward the brain) has higher oxygen saturation than does the blood in the descending aorta (toward the legs, Fig. 3). Additionally, the umbilical arteries carry some of the blood descending toward the leg back to the placenta.

**Table 1:** Neonatal and adult brain weight and total body weight for the great apes and human beings. Adult body weight is the average of male and female weight. Data from Harvey et al. [25]

	Neonatal mass (grams)			Adult mass (grams)		
Species	Brain	Body	Br/Bo ratio	Brain	Body	Br/Bo ratio
*Pongo* (orangutan)	170.3	1,728.0	0.10	413.3	53,000.0	0.008
*Pan* (chimpanzee)	128.0	1,756.0	0.07	410.3	36,350.0	0.011
*Gorilla*	227.0	2,110.0	0.11	505.9	126,500.0	0.004
*Homo sapiens*	384.0	3,300.0	0.12	1,250.0	44,000.0	0.284

This pattern of fetal circulation is common to most mammals and is likely to be evolutionarily ancient. Because energy and other growth requirements are limited, there is a trade-off between growth of the upper body and the legs, which results in the mammalian fetal pattern of body proportion of large head relative to short legs. To grow and maintain the larger human fetal brain, there is an amplification of the preferential flow of blood to the ascending aorta. The ancient circulatory pattern may leave the human fetal legs with a more acute reduced supply of oxygen and nutrients than other mammals, further slowing fetal leg growth and development compared with more cephalic regions of the body and compared with the fetuses of our smaller-brained primate cousins.

**Fig. 4:**
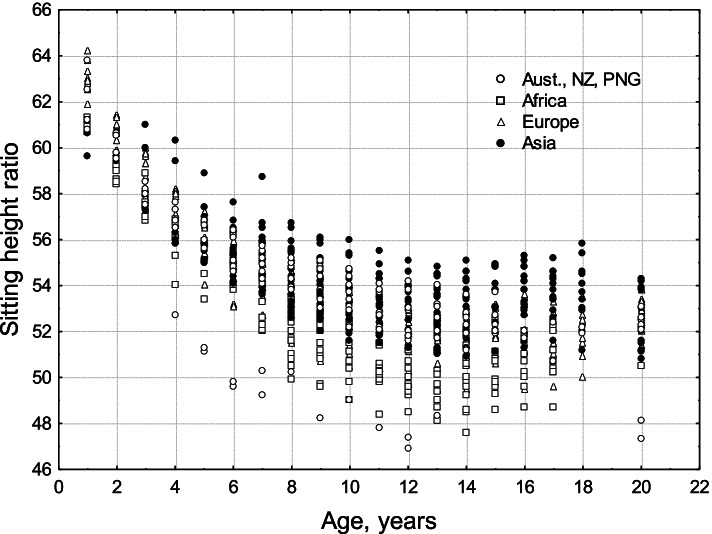
Sitting height ratio by age for the four geographic groups defined by Eveleth and Tanner [19, 20]. Age 20 includes data for adults over the age of 18 years. A larger SHR indicates relatively shorter legs for total stature. Original figure from [28]

Relative to total body length, boys and men tend to have longer legs and arms than girls and women, although much variation exists. Discrete populations of living humans present a diversity of body sizes and shapes. Mean stature for populations of adults varies from minimum values for the Efe Pygmies of Africa at 144.9 cm for men and 136.1 cm for women [26] to the maximum values for the Dutch of Europe at 184.0 cm for men and 170.6 cm for women [27]. There are also biologically and statistically significant variations between human populations in body shape. Eveleth and Tanner [19, 20] published data for height, body proportions, and leg length, estimated via the sitting height ratio (SHR), from dozens of human populations, distributed across most geographic regions of the world. Bogin and Rios [28] plotted the data for SHR to show that Australasians and sub-Saharan Africans tend to have longer legs and arms than Europeans and Asians (Fig. 4). Mean SHR for populations of adults varies from minimum values, i.e., relatively longest legs, for Australian First Nation people (identified as “Australian Aborigines” by Eveleth and Tanner) with a *SHR* = 47.3 for men and 48.1 for women, to the maximum SHR values, i.e., relatively shortest legs, for Guatemala Maya men and Peruvian women (*SHR* = 54.6 and 55.8).

## Applying Bergmann’s rule to human beings

Making sense of these worldwide comparisons is difficult because of the differences in lifestyle, environment, migration events, and genomics. Despite the plethora of possible factors, most anthropological, human biology, and human physiology research resorts to Bergmann’s and Allen’s rules as primary causes for the global patterns of human body shape variation.

Perhaps, the first researcher to carefully assess Bergmann’s rule as applied to humans was Derek Roberts, as mentioned above. He published an analysis showing a significant relationship between body mass and latitude, with groups of people living at higher latitudes having greater body mass than those living closer to the equator [16]. Twenty-five years later, Roberts [29] updated and reaffirmed these findings. Other research shows that people living in colder regions also tend to have shorter limbs relative to total stature, compared with groups of people living in warmer regions [30, 31].

**Fig. 5:**
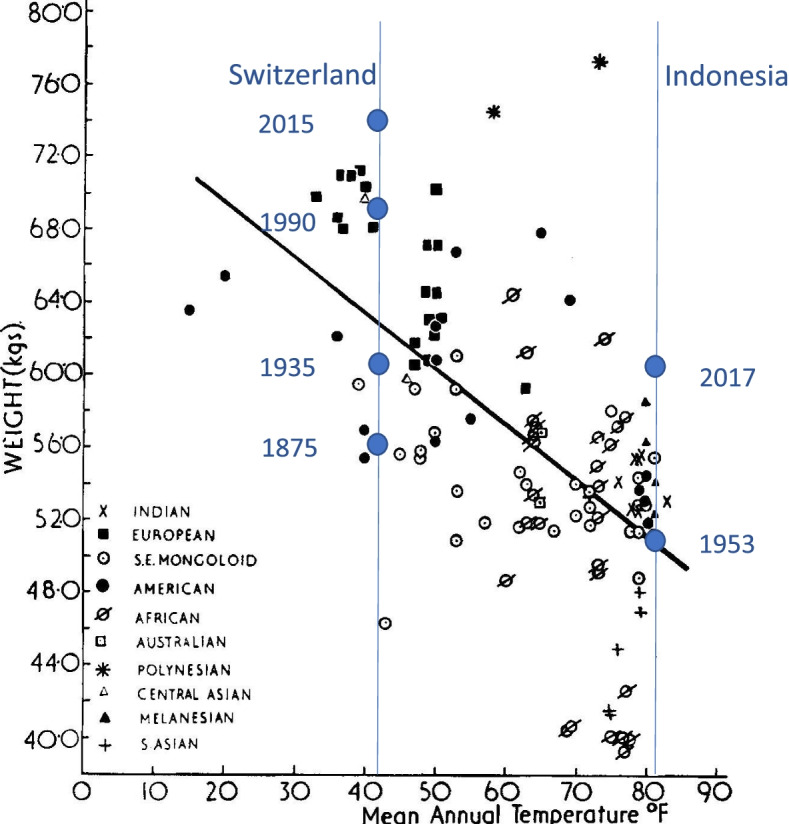
The relationship between mean annual temperature and body weight (mass) for the human groups analyzed by Roberts [16]. Superimposed on Roberts’ original illustration are body weight data for Swiss [32] and Indonesian men [33], all ~20 years old, measured in different years

In his famous compilation of studies, Roberts synthesized data from different ethnic groups living at mean annual temperatures between 10 and 80 °F and with adult men weighing between 40 and 77 kg. He found a negative relationship between body weight and mean environmental temperature with correlation value of *r* = −0.59. Robert’s 1953 illustration of the data is presented here as Fig. 5. This figure also includes data added by the present authors for Swiss and Indonesian samples, which are discussed below.

## Of cause and correlation

These climate relationships, however, are only correlations, with values in the range of *r* = −0.5 to −0.6. Correlations cannot prove causality or, even, the direction of association. It is, of course, absurd to claim that greater body mass of a mammalian species “causes” lower temperature. The reverse, however, is exactly Bergmann’s rule, but does this “rule” make a better explanation? In statistics, the square of the correlation value is often taken as an indication of the percentage of explained variance between two variables. Doing so for Bergmann’s rule means that climate “explains,” at maximum, about 36% of variation in human body mass. Other factors explain the other 64% of the variance.

A reanalysis of the Roberts’ data by Katzmarzyk and Leonard [34] modified the importance of climate as the primary molder of human body mass and proportion. Katzmarzyk and Leonard analyzed the SHR (which they called “relative sitting height”) of 165 human groups studied between 1960 and 1996. A larger SHR indicates relatively greater proportion of stature is due to the length of head, neck, and trunk of the body and/or shorter legs relative to total stature. Bergmann’s and Allen’s rules predict greater SHR in colder climates. The human data analyzed by Roberts were collected prior to 1953. Katzmarzyk and Leonard showed that the more recently studied groups still follow the ecological principles of body shape, but that the association with climate has been attenuated since Robert’s study. The slopes of the best-fitting linear regression lines for the relation of mean annual temperature to SHR were half those reported by Roberts, that is, the correlation between body and mean annual temperature declined to *r* = −0.3 or less in both sexes. Statistically, the variance explained has dropped to only 9%, which is not a biologically meaningful percentage. Similar findings were reported by Foster and Collard [35] based on a global sample of 263 human groups, “…believed to have resided in their present location since 1492” (p. e72269). The data were collected during the twentieth century. These authors used linear regression to estimate the relationship between absolute latitude and mean annual temperature with body mass, body mass index (kg/ht^2^), the ratio of surface area to body mass (SA/BM), and the ponderal index (kg/ht^3^). The percent of variance explained (the R^2^ values) in these associations varied from a minimum of 0.07 for latitude and ponderal index to a maximum of 0.33 for latitude and body mass. Most of the other R^2^ values were ~0.20. Again, these values have little biological meaning.

The mean annual temperature data for different latitudes used in the analysis by Katzmarzyk and Leonard were taken from sources published between 1970 and 1981. Today, we know that there was a trend of mean global temperature increase during the twentieth century, by about +0.35 °C between 1950 and 1990. Katzmarzyk and Leonard did not consider this trend, and it is just as well that they did not. It is unlikely that the small rise in average global temperature can account for the reduced correlation between body shape and temperature across some 20–30° of latitude between arctic and tropical climates. Katzmarzyk and Leonard reported that the primary reason for the lower correlation was the increase in body mass of the tropical latitude populations, that is, from those regions of the world with a mean average annual temperature ≥ 15 °C. The authors proposed that changes in nutrition and health were the likely reasons for the rapid increases in body mass. Subsequent research confirms the epidemic rise of overweight and obesity of tropical latitude peoples around the world, especially in the current lower-income nations that were colonial era territories of Europe and the USA [36, 37]. Katzmarzyk and Leonard, as well as the more recent research, identified several nutritional and lifestyle changes, such as the introduction of western foods, improved health care, and reduced physical activity, which promote greater body mass, especially greater fatness. These same nutrition, health, and lifestyle factors may increase stature and especially increase leg length relative to total stature, as has been shown for Maya boys and girls raised in the USA versus same-age Maya in Guatemala [28, 38]. Katzmarzyk and Leonard's conclusion was that global phenotypic variability in body proportions is not primarily due to temperature; rather, “…the findings presented here suggest that there is a significant developmental component to [sitting height ratio], which is shaped by the influence of nutrition and other environmental parameters…” (pp. 493-4). They also emphasized that there is a “significant developmental component” to adult body mass and surface area. According to Katzmarzyk and Leonard, and many other researchers [24, 39, 40], during the years of growth and development, the intake of more or less food, more or less of any essential nutrient, and more or less physical activity (and the type of activity) could influence body shape, body size, and body composition (fatness and muscularity).

The hypothesis that food more than temperature underlies Bergmann’s rule was proposed previously by Geist [8], who stated in the title of his article that “Bergmann’s rule is invalid.” Geist proposed that the correlation of body mass with temperature is spurious. Underlying the relationship between temperature or latitude and body size was the duration of “…the annual productivity pulse…,” defined as food availability per animal during the growing season. Geist summarized his analysis by writing “…that body size is a function of availability of nutrients and energy during periods of growth. Correlations between body size and temperature are shown to be spurious. If reduction in relative surface area is indeed an adaptation to conserve heat, then mammals should increase in size from south to north at rates two orders of magnitude greater than they do. Bergmann's rule has no basis in fact or theory” (p. 1035).

Building on these previous analysis, Pomeroy and colleagues [11] analyzed global variation in stature, sitting height, and absolute and relative lower leg length (LLL) using global samples of data from 571 groups of adult men and 268 groups of adult women. The anthropometric variables were assessed in relation to temperature, humidity, and net primary productivity (NPP), which is equivalent to Geist’s “annual productivity pulse.” The authors also estimated a variable called “population history,” which was modeled as the “…geographic distances reflecting the hypothesized pattern for the spread of modern humans out of Africa” (p. 1). How “population history” influences body size and shape is not explained by the authors, and its meaning is left to the reader’s imagination (might it relate to genetics, migration history including forced movement due to the slave trade or warfare, or something else?). The authors used linear regression analysis and reported that population history and NPP explained more variation in stature, sitting height, and LLL than the climate variables. The significant climate variables were consistent with Allen’s rule but not Bergmann’s rule.

## Serendipity versus science

The belief in the fundamental truth that Bergmann’s rule applies to humans is based on the accidental association of height, weight, and average temperature variation by latitude that existed at the time of Robert’s analysis. Before and after that time, that is, before and after 1953, the situation was different, and Bergmann’s rule did not apply. Roberts was, in a sense, lucky when he did his 1953 analysis because he was really describing the effect of secular trends for increased height in the rich northern nations versus the stagnation or decline in height of people in the lower-income colonial era southern nations. In 1953, this difference in average adult stature was at its maximum. The height difference had everything to do with differences in the standard of living and the quality of the social-economic-political-emotional (SEPE) environment [41]. The differences had nothing to do with temperature or climate.

During the years between Bergmann’s work and Roberts’ compilation, average body height of populations of many nations, and particularly of the industrialized countries, significantly increased. In a meticulous collection of global height changes between the mid-nineteenth and the mid-twentieth century, Kenntner [42] showed that the European populations had on average increased in height by 8 to 10 cm, and the Netherlands by 15 cm, since 1850. Similar increases in adult height had not been observed in the tropical European colonies. A recent analysis on a century of trends in adult human height confirmed that there was a little gain in average height in the countries of sub-Saharan Africa and South Asia at the time of Roberts’ compilation [43]. The body height gap between countries of the northern hemisphere and their tropical colonies was considerable. This changed in recent decades as adult body height significantly increased since the independence of many former European colonies. Changes in mean weight over time for two nations, Switzerland and Indonesia, are shown in Fig. 5. The Swiss data illustrate a “long secular trend” of 140 years and the Indonesia data a “short secular trend” of 64 years. In the year 2009, the Indonesians had about the same body mass as the Swiss of 1935. Latitude did not change, and adaptation to temperature is irrelevant to these long and short secular changes.

As discussed above, in 1998, Katzmarzyk and Leonard [34] published post-1953 data on the relationship between body weight and mean annual temperature of their habitats. In a further update, Leonard summarized that “…the correlation between mass and temperature was much lower in later samples, and the slope of the regression was significantly shallower than that reported by Roberts” [44]. Leonard concluded that “…these differences partly reflect secular changes in growth and body size, and the development of improved technology that moderates extreme temperature exposure…[and that]…these findings underscore the importance of both nutritional and temperature stresses in shaping human variation in body size and shape” ([39], p. 816). These statements are certainly intuitive but are symptomatic of human thinking regarding traditional knowledge, of which Bergmann’s rule is a conspicuous example. It is not Bergmann’s rule itself that is questioned; rather, the weakening of the association between size and temperature is ascribed to changes in those factors which, according to the rule, are believed to influence body height and weight.

## Stature is a not a synonym for nutrition or of temperature

Short or tall adult stature can no longer be considered a simple synonym of nutrition history. It is shown in several recent analyses that height variation within several equatorial and temperate latitude groups of people is quite independent of diet (see especially [45] and others [46–48]). Starvation will certainly inhibit height growth, but the relatively short stature of, for example, some Indonesian children is unrelated to food availability, body weight, fatness, and physical performance. Rather, the short stature of these children, many of whom are classified as “stunted” by international nutrition/growth references, is better explained by having parents with poor education and by the families suffering social disadvantage [49]. We explain below how disadvantage and other non-nutritional factors regulate body size and shape. Nor is the increase in height, body mass, and fatness of recent Europeans, and others around the world, explicable by outdoor or indoor domestic temperature. Quite in contrast, these temperatures have significantly increased over the past century or more and, thus, according to Bergmann’s rule, should have rather resulted in a decline instead of a further elevation of body size in the northern Europeans [50].

## The complex regulation of body size and shape

Living under extreme geographic circumstances, such as cold, heat, high altitude, or high latitude, exerts evolutionary pressure that those who live in other regions do not experience. In response, classic evolutionary forces, such as mutation and natural selection, may act on human phenotypes. In his 2018 review article, Leonard summarized several mutations being associated with enhanced fatty acid oxidation that are prevalent in northern populations. Yet, size is a complex trait. Bergmann himself realized that by far, not all arctic species are larger than their equatorial counterparts. Since Bergmann’s time, human biologists and anthropologists have come to appreciate the importance of developmental plasticity in human growth as the primary regulator of size and shape [24, 51–53].

Katzmarzyk and Leonard [34] emphasized the impact of rapid changes to diet, health, and lifestyle on the body size, shape, and composition (muscularity versus fatness) on tropical latitude peoples. It is known that these rapid changes interact in complex ways with growth and development of human phenotypes. Furthermore, it is known that diet, health, and lifestyle are influenced by a complex matrix of factors from the social-economic-political-emotional (SEPE) environment. An explanation of physiological mechanisms by which SEPE factors regulate plasticity in human growth and development was published by Bogin [41]. Here is a provided summary, with examples, of the impact of SEPE factors on mammalian, including human, phenotypes.

## Social-economic-political-emotional (SEPE) factors influence body size and shape

Recent research with social mammals shows that the regulation of growth does not follow some simple genetic program but is strongly regulated by SEPE factors [41, 54, 55]. A growth pathway from parental social status to offspring body size is a well-tested hypothesis that has been empirically documented in several nonhuman species, such as baboons [56], mandrills [57], orangutans [58–60], mole rats [61], meerkats [55, 62, 63], and other species [54]. The importance of SEPE factors in each of these cases is that all of these mammals live in *social* groups where access to critical resources (*economic*), such as nesting space, food, and mating partners, is influenced by the dominance hierarchy (*political*) and/or degree of aggressiveness versus affiliation (*emotional*) of individuals within the group. In humans, growth and final height appear to have causal associations to peer groups, social networks, and dominance within the group [64–69]. Human societies, of course, are different from the nonhuman social mammals due to culture, especially the ideological justifications for social-economic-political dominance of the elites. These ideological justifications are today enshrined in the hereditary aristocracies, constitutional monarchies, parliamentary monarchies, taxation laws, and other forms of anti-egalitarian status differentials practiced in many of the wealthier North American, European, and Asian nations. The elites know that they are superior and are treated as such by the non-elites. In all human societies, the elites are, on average, taller than lower social-economic-political classes [24].

Improvements in SEPE factors leading to social mobility, higher wages, greater democratization, and greater feelings of security have given rise to the exuberant increase in height within a few generations in the contemporary industrial world. The shortness in height currently observed in the low- and middle-income countries may indicate their delay in SEPE modernization when compared to the Western world, but it must not be mistaken as an example of Bergmann’s rule. There is no evidence that Bergmann’s rule applies in these studies of present-day humans.

Elsewhere [41, 70], additional evidence and biological mechanisms are provided, that is, associate gradients in height between elites and non-elites with physical and emotional stimulation operating through SEPE networks. Differences between the networks provide positive stimulation for the elites and negative stimulation for the lower classes that explain much of the downward gradient in height from the elites, to the middle classes, and finally to the lower classes of people within a given society.

## Change of political climate and the plasticity of human body

The phrase “political climate” is used here as an analogy and contrast to the meteorological climate. This example describes the political weather of the last 35 years in Germany. This was a period of notable modification of anthropometric parameters during the breakup of the former Soviet Union and the transition of the political, socioeconomic, and emotional circumstances. There was a stark contrast in the economic and political situation between the former German Democratic Republic (GDR, i.e., East Germany) and West Germany prior to reunification and during the early 1990s. At the time of reunification in 1989, the body height of school children of East Germany was significantly shorter than that of same-age school children in West Germany, but the difference nearly disappeared within 4–5 years. Pelvic and elbow breadth of all the children decreased at the beginning of 2000, and their fat distribution pattern became more “feminine” after 1997, that is, relatively more fat on hips and lower extremities [71]. The increase of height was likely due, in part, to SEPE factors, especially the community effect on growth [66, 72, 73] following the political reorganization of the former East Germany. The community effect hypothesis predicts that that there are influences on the attainment of final height, weight, body composition, and body proportions which arise from the biosocial-psychological proximity of members within a social network. Two of the major factors regulating community effects are emotions related to perception of socioeconomic status and to ego motivation. These emotions (feelings and thoughts) are transduced in the brain and influence production of growth hormone, insulin-like growth factor-1, and other endocrine hormones [24]. As new generations of children from the former two Germanys grew up together after reunification, they came to share greater and greater similarity in SEPE climates, lifestyle, and aspirations for the future. Their socioeconomic status became more equal, they had greater ego motivation for success in the society, and their body size and shape became more homogeneous. This phenotype plasticity had nothing to do with temperature or Bergmann’s rule.

Two additional examples add support to the interpretation that population variation in body shape is primarily related to nutritional and SEPE influences, but not to temperature. The first is based on studies conducted since the 1960s with Guatemala Maya people. The rural Maya often lives at altitudes above 1500 m and experience cold temperatures. Their short stature with particularly short legs has been cited as an example of Bergmann’s rule. It was known, however, that the rural Maya consumed only about 80% of the total energy needed for healthy growth, and most of the population suffered much infectious disease and intestinal parasites. In addition, 20.4% of primary school children were also iodine deficient [28]. Iodine deficiency during infancy and childhood results in reduced leg length growth, especially at the epiphyses of the distal femur, the tibia, and the foot. Maya children and adult participants in those studies spent considerable time and energy at heavy labor, which diverted available energy in the diet away from growth. This nutrition and lifestyle combination is known to reduce total stature, especially leg length. The second example comes from similar findings reported for native Peruvian highland children of the Andes mountains by Emma Pomeroy and colleagues [74]. Pomeroy’s research group reported trade-offs in relative limb length. Those children exposed to greater nutritional deficiencies and work had significantly shorter limbs, hands, and feet compared with less stressed lowland children. The more stressed participants also lived at high altitude and suffered more cold stress. In their 2021 update, Pomeroy and colleagues reported that coldness due to altitude had no independent effect on their analysis of body shape. The researchers also showed that differences between the groups in head-trunk length were smaller. The ulna and tibia bones were the most sensitive to stressful nutritional and SEPE environment conditions.

These examples emphasize that the small body size and body shape of the Maya and native Peruvians is an unhealthy response to dietary and infectious disease stress and poor SEPE conditions. These examples negate Bergmann’s rule. It is clear from these examples that body size and shape are not beneficial adaptations to the temperature climate. Maya and native Peruvians suffer from an unhealthy emotional climate due to insecurities in education, health care, employment, and housing. The insecurities are associated with adverse childhood experiences and chronic toxic stress that are known to delay skeletal growth, especially in the legs [70].

## Conclusion

Carl Bergmann was an astute naturalist and physiologist. His ideas about animal size and shape were important advances in the pre-Darwinian nineteenth century. Today, Bergmann’s rule is a “just-so” story and should be relegated to teaching and scholarship about the history of science. That “rule” is no longer acceptable science and has nothing to tell us about physiological anthropology.

## Data Availability

Data are available from the authors for those studies conducted by the authors.

## Footnotes

1 This is the translation from the original German language in which the book was published. All other quotations used here are translations from the original German.

2 Lieschen Müller is a placeholder for the average person in the German-speaking world. The average is often also referred to as “Lieschen Müller.” German parents tell their children Lieschen Müller stories to explain how the world works.
